# MCFNet: Multi-Scale Contextual Fusion Network for Salient Object Detection in Optical Remote Sensing Images

**DOI:** 10.3390/s25103035

**Published:** 2025-05-12

**Authors:** Jinting Ding, Yueqian Quan, Honghui Xu

**Affiliations:** 1 School of Information and Electrical Engineering, Hangzhou City University, Hangzhou 310015, China; dingjt@hzcu.edu.cn; 2 School of Computer Science and Technology, Zhejiang University of Technology, Hangzhou 310023, China; qyq@zjut.edu.cn

**Keywords:** salient object detection, optical remote sensing image, contextual interconnection, semantic-aware attention

## Abstract

The rapid advancement of deep learning has catalyzed progress in salient object detection (SOD), extending its impact to the domain of optical remote sensing images (ORSIs). Despite increasing attention, salient object detection for optical remote sensing images (ORSI-SOD) remains highly challenging due to the intrinsic complexities of remote sensing scenes. In particular, severe variations in object scale and quantity, cluttered backgrounds, and irregular object morphologies significantly hinder accurate target localization and boundary delineation. In response to these challenges, we introduce the Multi-scale Contextual Fusion Network (MCFNet) for ORSI-SOD. MCFNet incorporates a Semantic-Aware Attention Module (SAM), which provides explicit semantic guidance during feature extraction. By producing preliminary semantic masks, SAM enables the network to capture long-range contextual dependencies, thereby enhancing localization accuracy for salient objects exhibiting substantial scale variation and structural complexity. In addition, MCFNet integrates a Contextual Interconnection Module (CIM), which promotes effective fusion of local and global contextual features. By facilitating cross-layer interactions and adopting a multiscale refinement strategy, CIM enriches texture representations while suppressing background interference, leading to smoother object boundaries and more precise delineation of salient regions. Extensive evaluations conducted on three standard ORSI-SOD benchmark datasets demonstrate the superior performance of MCFNet compared to existing methods, highlighting its robustness and efficiency in handling challenging remote sensing scenarios.

## 1. Introduction

Salient Object Detection (SOD) [1] is a fundamental task in computer vision, aimed at emulating the human visual system’s ability to identify and localize the most visually prominent regions within a scene. In recent years, SOD has found widespread applications in various domains, including image segmentation [2,3], image quality assessment [4], visual target tracking [5,6], and content-aware image editing [7]. Optical Remote Sensing Images (ORSIs) are captured by satellites and onboard antenna sensors. Salient object detection in remote sensing images (ORSI-SOD) [8] can effectively identify the most salient target areas in ORSIs with the help of in-depth learning and detailed analysis of image features in both spatial and spectral dimensions. However, the ORSI-SOD task faces several challenges due to the high-altitude and long-distance nature of image capture. In particular, the variability in target numbers and scales is a significant issue, as a single image may contain numerous targets of varying sizes. Furthermore, the complexity of the background environment poses further challenges, as it can obscure the detection of salient targets. Irregular topological features within ORSIs also contribute to the difficulty of accurately identifying prominent targets.

Recent years have witnessed notable advances in salient object detection for natural scene images (NSI-SOD), leading to the development of numerous effective methods. These methods can be broadly divided into main types: traditional methods and deep learning-based methods. Traditional methods [9,10] rely primarily on model-driven feature extraction [11] and classical machine learning algorithms [12]. Although grounded in solid theoretical foundations, these approaches frequently encounter difficulties in delivering precise detection results when faced with intricate or cluttered scenes because of limited generalization capabilities and poor adaptability to diverse background conditions. In contrast, the advent of convolutional neural networks (CNNs) has led to the development of data-driven deep learning models [13,14] that have demonstrated remarkable performance in NSI-SOD tasks. These approaches leverage large-scale annotated datasets to learn robust and discriminative feature representations. For instance, the PA-KRN [15] adopts a hierarchical architecture to enhance salient object detection in natural images, while the SUCA [16] further improves multiscale target perception through stacked interaction networks. Such deep learning methods have consistently outperformed traditional methods in accuracy and robustness. However, directly applying NSI-SOD models to ORSI-SOD tasks is not trivial [17]. ORSIs differ markedly from NSIs in terms of spatial distribution, viewpoint, resolution, and overall scene composition. In addition, they exhibit greater variability in object scale, shape, and appearance. These domain-specific challenges limit the effectiveness of NSI-SOD models when transferred to remote sensing scenarios. As a result, there is a critical need for the development of specialized and adaptive methods tailored to the unique demands of ORSI-SOD.

In response to the limitations of transferring NSI-SOD methods to remote sensing scenarios, several approaches [18,19] specifically designed for ORSI-SOD have been proposed. For example, nested networks [20] generate high-level attention maps by integrating low-level cues with deeper features through a dense attention mechanism. However, these methods often overlook the impact of high-level semantic information on low-level feature representation during attention transfer, which compromises the refinement of object boundaries. ACCoNet [21] improves saliency detection by enabling cross-layer feature interaction and effectively extracting contextual semantic information. Despite its effectiveness in contextual modeling, it incurs substantial computational overhead. AESINet [22] employs separate modules to process high- and low-level features extracted from multiple CNN layers and captures adaptive edge information through perceptual mapping of self-pooled differences. Although these approaches are specifically tailored for ORSI-SOD, they remain limited when confronted with the compounded challenges of varying object quantities and scales, intricate background environments, and irregular object topologies.

Motivated by the limitations of existing approaches, we aim to tackle the persistent challenges in ORSI-SOD, such as background clutter, variable object scales and densities, and inherently low contrast between targets and their surroundings. In this study, we introduce a novel Multi-scale Contextual Fusion Network (MCFNet), which is specifically engineered to achieve robust and accurate saliency detection under complex and diverse conditions. MCFNet is constructed based on two complementary modules: the Semantic Attention Module (SAM) and the Contextual Interconnection Module (CIM). SAM plays a pivotal role in precise target localization. It integrates coarse semantic masks as initial priors, providing robust and explicit semantic guidance to subsequent layers. In contrast to conventional methods, which often falter in capturing long-range dependencies, SAM introduces a global semantic perception mechanism. This mechanism effectively overcomes the constraints imposed by local receptive fields, allowing the network to accurately localize salient regions, even when dealing with objects of varying scales and spatially dispersed distributions. Working in tandem with SAM, CIM is crucial for enhancing object saliency and suppressing background noise. Given that RSIs frequently contain background elements that resemble foreground targets in texture or color, the risk of false positives remains high. CIM addresses this issue by employing a multiscale refinement strategy, enabling deep, cross-layer feature interactions. Through the mining of inter-scale contextual dependencies, CIM effectively integrates high-level semantic cues with low-level texture details. To comprehensively evaluate the effectiveness of MCFNet, we perform extensive experiments on three widely used public ORSI-SOD benchmarks. Across all datasets and most evaluation metrics, MCFNet consistently outperforms 20 state-of-the-art methods, demonstrating clear superiority in both detection accuracy and robustness.

The main contributions of this work can be outlined as follows.

1. We introduce a Multi-scale Contextual Fusion Network (MCFNet) built upon an encoder–decoder framework, designed to effectively extract high-level semantic cues while promoting the integration of low-level detailed features with high-level contextual information via a cross-level interconnection strategy.
2. We introduce a Semantic-Aware Attention Module (SAM), which leverages preliminary semantic masks to guide the extraction of deep semantic information in ORSIs, thereby enhancing the ability to localize salient targets under complex conditions.
3. We design a Contextual Interconnection Module (CIM) that enriches texture representations across multiple scales by refining local features and adaptively fusing contextual information from adjacent layers.
4. Comprehensive evaluations conducted on three datasets, ORSSD, EORSSD, and ORSI4199, show that our approach achieves superior performance compared to existing methods. Furthermore, ablation studies confirm the contribution and effectiveness of each proposed module.

## 2. Related Work

### 2.1. Salient Object Detection Methods in NSIs

The SOD task was originally developed for NSIs analysis. Over the past few years, NSI-SOD has witnessed substantial progress, with a wide range of methods proposed, spanning both traditional and deep learning approaches. Traditional NSI-SOD approaches are generally grouped into three categories: unsupervised methods [11,23,24,25], semi-supervised methods [26,27], and supervised methods [28]. Among these, unsupervised methods have historically dominated, typically relying on interpretable handcrafted features extracted through image preprocessing techniques, such as the sparse graph [11], the saliency tree [23], the maximal entropy random walk [24], and the regularized random walks ranking [25]. In contrast, relatively few traditional methods adopt semi-supervised or supervised strategies. For instance, Zhou et al. [26] developed a boundary-guided approach for pseudo-label generation and designed a semi-supervised learning framework based on a linear feedback control mechanism for iterative optimization. Liang et al. [28] applied supervised learning via support vector machines to select salient features and eliminate redundancies, thereby accelerating model training. Despite their contributions, traditional NSI-SOD methods struggle to generalize across the diverse and complex scenarios encountered in ORSIs.

CNN-based methods for NSI-SOD have overcome many of the limitations inherent in traditional approaches. Unlike traditional techniques, most CNN-based methods adopt a supervised learning paradigm, relying on large-scale annotated datasets to significantly enhance detection accuracy. Over the past years, several influential feature processing strategies have been introduced, including deep supervision [29], multi-scale interaction [14,30], and edge-aware feature enhancement [13]. Huang et al. [29] were among the first to incorporate deep supervision into NSI-SOD frameworks, inspiring a wave of subsequent research exploring its potential. PoolNet [30] utilizes a combination of top-down and bottom-up pathways to extract and integrate multi-scale contextual information. GateNet [14] introduces collapsed spatial pyramid pooling to aggregate spatial feature maps at varying receptive fields, thereby enhancing multi-scale representation. Meanwhile, EGNet [13] focuses on capturing edge features, improving the precision of object localization by highlighting object boundaries more effectively. Although deep learning-based NSI-SOD methods have achieved remarkable success, applying them directly to ORSI-SOD tasks poses considerable challenges. The fundamental differences between NSIs and ORSIs pose substantial challenges, limiting the generalizability and effectiveness of NSI-SOD models in remote sensing contexts.

### 2.2. Salient Object Detection Methods in ORSIs

Compared with the NSI-SOD task, ORSI-SOD faces more complex and unique challenges. To tackle these challenges, CNN-based models for ORSI-SOD offer promising solutions owing to their strong abilities in feature representation and learning. Specifically, Ma et al. explored efficient segmentation solutions for SAR images from different technical perspectives, proposing a fast segmentation method based on deep task-specific superpixel sampling and soft graph convolution [31] as well as a task-specific region merging strategy for rapid segmentation [32]. Li et al. [20] designed an LV-shaped network architecture, where an L-shaped pyramid module processes inputs at different resolutions, and a V-shaped nested connections structure precisely identifies the position and boundaries of salient objects. Luo et al. [33] further enhanced feature learning by adopting convolution kernels of diverse shapes, which enables it to capture a wide range of salient target features, including topology and orientation, thereby improving the model’s adaptability to complex target morphologies. Tu et al. [34] combined local features extracted via pyramid pooling with global context to construct a strong multi-scale regional representation. Gu et al. [35] introduced an edge-guided branching structure to generate rich boundary information, leading to accurate localization and highlighting of salient regions under boundary label supervision. Despite the remarkable progress made using deep neural networks in ORSI-SOD, CNN-based models face inherent limitations in global modeling capabilities, which often results in difficulty detecting objects across varying scales simultaneously. This problem is especially prominent in RSIs, which usually contain richer and more diverse scales and target morphologies, putting forward higher requirements on the global perception ability of the model.

Transformers [36] excel at capturing long-range interactions, enabling effective extraction of global context from visual data. Consequently, recent studies have increasingly incorporated Transformers into the ORSI-SOD task to enhance global modeling capabilities. Specifically, Zhang et al. [37] designed a dense attention flow structure, which facilitates the efficient connection and fusion of features at multiple levels through an attention mechanism. Zhou et al. [38] introduced a U-shaped architecture, incorporating edge-aware positional attention units to strengthen edge cue extraction, thereby improving the detection accuracy of salient targets. Inspired by this, we propose an efficient global–local perception network based on contextual interactions. Within this network, global information, which is extracted through semantic-aware attention mechanisms, is progressively integrated with local texture details via contextual interactions at every layer. This novel architecture enhances the model’s ability to model complex global dependencies while enabling accurate localization and refinement of fine-grained local features.

## 3. Method

**Figure 1:**
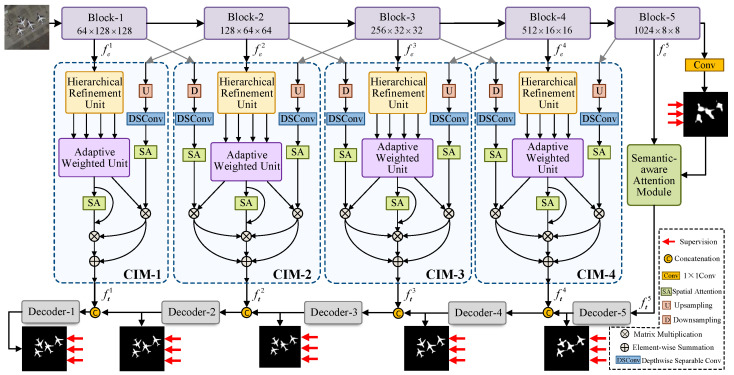
The proposed Multi-scale Contextual Fusion Network (MCFNet) for ORSI-SOD, which is structured around two core modules: the semantic-aware attention module (SAM) and the contextual interconnection module (CIM).

In this study, we present a comprehensive analysis of the proposed Multi-scale Contextual Fusion Network (MCFNet), as shown in Figure 1. MCFNet adopts an encoder–decoder framework and integrates two key components: the Semantic-aware Attention Module (SAM) and the Contextual Interconnection Module (CIM). In Section 3.1, we provide a brief overview of the MCFNet framework. Subsequently, in Section 3.2 and Section 3.3, we offer an in-depth discussion of these two core components. Finally, in Section 3.4, we elaborate on the design of the decoder and the hybrid loss function.

### 3.1. Network Overview

Figure 1 presents the architecture of MCFNet, which adopts a generalized encoder–decoder framework comprising an encoder, a semantic-aware attention module (SAM), a contextual interconnection module (CIM), and a decoder. For feature extraction, we employ Res2Net-50 [36] as the backbone. The encoder is structured into five blocks, which capture multi-scale features encompassing both low-level and high-level information. The output of each block can be represented as $f_{e}^{i}\in R^{C_{i}\times H_{i}\times W_{i}}$, i∈{1,2,3,4,5}, where $H_{i}$ and $W_{i}$ are $\frac{256}{2^{i}}$ and $C_{i}\in \left\{64,128,256,512,1024\right\}$. Notably, the high-level feature encapsulates rich semantic information crucial for ORSI-SOD. For high-level feature $f_{e}^{5}$, we employ SAM, which enhances semantic detail and suppresses background noise, thereby improving the localization of salient regions in complex scenes. In contrast, the low-level features $f_{e}^{1}$, $f_{e}^{2}$, $f_{e}^{3}$, and $f_{e}^{4}$ possess abundant texture information but lack global context. To address this, the CIM aggregates cross-scale features from adjacent blocks, facilitating enhanced interaction between low-level and high-level representations. Finally, the decoder consists of five blocks that progressively reconstruct the resolution. The hybrid loss function is employed to enable deep supervision and produce the final output prediction maps.

### 3.2. Semantic-Aware Attention Module

When processing remote sensing images, salient objects exhibit substantial variability in number and scale and are often obscured by complex backgrounds, presenting significant challenges for accurate target identification and localization. To address these issues, most CNN-based methods typically employ deep convolutional stacks to extract features from increasingly large receptive fields. However, these methods tend to lose critical target information during progressive downsampling, largely due to the inherent limitations of CNNs in global context modeling. Although CNNs effectively capture local image content, their extracted features are insufficient to suppress background noise and achieve precise target localization. In contrast, the self-attention mechanism has gained recognition for its superior ability to model global dependencies [39]. Motivated by this insight, we propose the Semantic-aware Attention Module (SAM), which leverages a preliminary semantic mask to guide feature extraction. SAM enhances critical global information, thereby facilitating robust and accurate target localization by efficiently capturing long-range dependencies and overcoming the limitations of local feature extraction.

**Figure 2:**
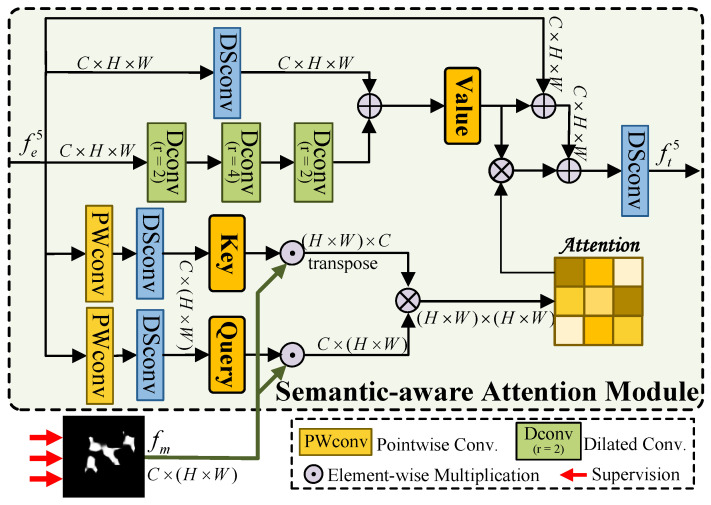
Illustration of semantic-aware attention module (SAM).

The high-level feature $f_{e}^{5}\in R^{C\times H\times W}$ extracted from the encoder’s fifth layer contains essential semantic information crucial for object detection. To enhance the localization of objects, we first process $f_{e}^{5}$ through a series of operations—including a 1×1 convolution, batch normalization, ReLU activation, and sigmoid function—to generate a preliminary semantic mask $f_{m}$. In this process, we preserve the preliminary semantic mask as continuous values within the [0,1] interval rather than binarizing them. Subsequently, we input the preliminary mask $f_{m}$ along with the original $f_{e}^{5}$ into our proposed SAM. As depicted in Figure 2, within the SAM, a bilinear fusion scheme is first employed to compute the attention matrix. Specifically, the input feature $f_{e}^{5}$ is efficiently decomposed into two intermediate features $x_{1}\in R^{1\times H\times W}$ and $x_{2}\in R^{1\times H\times W}$ via pointwise convolution. Then, the depthwise separable convolution layer is used to generate a query vector $Q\in R^{C\times (H\times W)}$ and a key vector $K\in R^{C\times (H\times W)}$. This effective combination of pointwise and depthwise separable convolutions preserves powerful feature extraction while significantly reducing computational complexity. To emphasize salient targets in the remote sensing image, we apply the preliminary semantic mask $f_{m}$ to both *Q* and *K* via element-wise multiplication. This operation accentuates the target region while suppressing extraneous information, yielding the semantic-aware query $Q^{\prime }$ and key $K^{\prime }$, which can be formulated as:

(1) $$\begin{array}{cc} \; & Q^{\prime }=f_{m}⊙DSconv\left(PWConv\left(f_{e}^{5}\right)\right), \end{array}$$

(2) $$\begin{array}{cc} \; & K^{\prime }={\left(f_{m}⊙DSconv\left(PWConv\left(f_{e}^{5}\right)\right)\right)}^{T} \end{array}$$

where DSConv(∗) is the depthwise separable convolution, PWConv(∗) is the pointwise convolution, and ⊙ represents the element-wise multiplication. Subsequently, $Q^{\prime }$ and the transposed $K^{\prime }$ undergo matrix multiplication to produce an attention map $A\in R^{\left(H\times W)\times (H\times W\right)}$, which quantifies the association strength between different spatial locations. The computational results are presented below.

(3) $$A=Softmax(Q^{\prime }⊗K^{\prime })$$

where ⊗ is the matrix multiplication, and Softmax(∗) denotes the softmax layer. Based on these operations, the resulting attention matrix identifies effectively encoded spatial information from a global perspective, thereby capturing and leveraging semantic cues.

To construct the value matrix *V* in the self-attention mechanism, we apply a depthwise separable convolution layer to the high-level feature $f_{e}^{5}$ to efficiently refine the target feature representation. Owing to its inherently small receptive field, depthwise separable convolution effectively emphasizes local feature details. In parallel, feature $f_{e}^{5}$ is processed through three sequential dilated convolution layers with expansion rates of 2, 4, and 2, respectively, to broaden the receptive field and capture long-range contextual information. By integrating these two strategies, the value matrix *V* simultaneously captures local details and global context, which is computed as:

(4) $$V=DSConv\left(f_{e}^{5}\right)⊕DConv_{r=2}\left(DConv_{r=4}\left(DConv_{r=2}\left(f_{e}^{5}\right)\right)\right)$$

where ⊕ represents the element-wise summation and $DConv_{r=2}$ and $DConv_{r=4}$ denote dilated convolutions with dilation rates of 2 and 4. In the final stage, we multiply the attention matrix *A* with the value matrix *V* and obtain the semantic-aware feature vector $f_{s}^{5}$. Then, the semantic-aware feature $f_{s}$ is fused with the original feature vector $f_{e}^{5}$ to yield the final output $f_{t}^{5}$ of SAM, which can be formulated as follows:

(5) $$f_{t}^{5}=DSConv\left(f_{e}^{5}⊕V⊕\alpha \cdot \left(A⊗V\right)\right)$$

where α is a learnable scaling parameter. Within the SAM, we employ a semantic-aware attention mechanism designed to amplify the salient object’s semantic representation while attenuating noise interference, thereby enabling high-precision target localization.

### 3.3. Contextual Interconnection Module

By employing SAM, we improve salient object localization by emphasizing global semantic cues. However, it may overlook fine-grained details essential for precise segmentation. In contrast, low-level features $f_{e}^{1},f_{e}^{2},f_{e}^{3},$ and $f_{e}^{4}$ encode high-resolution textures and subtle spatial details crucial for delineating accurate object boundaries. Recognizing that effective segmentation requires a balanced interplay between semantic-rich high-level context and detailed low-level features, we propose the Contextual Interaction Module (CIM). CIM integrates adjacent-level features, facilitating multi-scale information interaction via a dedicated Hierarchical Refinement Unit, which restores detailed feature representations. Additionally, an adaptive weighted unit is introduced to further enhance local feature representations, contributing to improved segmentation precision and stability.

**Figure 3:**
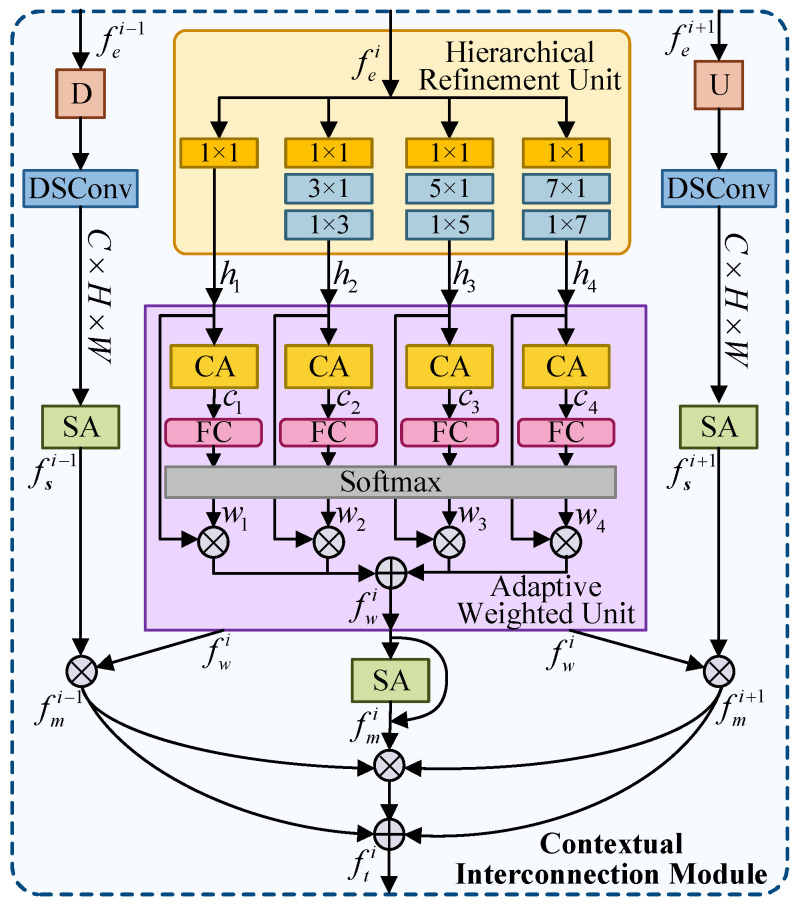
Illustration of contextual interconnection module (CIM).

In Figure 3, CIM comprises three primary branches, CIM-2, CIM-3, and CIM-4. Each of these branches integrates contextual information derived from its corresponding local and adjacent feature scales. Notably, CIM-1 incorporates only two branches. Accordingly, the operation of CIM can be abstracted as a generalized function F(∗), which can be formulated as follows:

(6) $$f_{t}^{i}=\left\{\begin{array}{cc} F(f_{e}^{i},f_{e}^{i+1}), & i=1\\ F(f_{e}^{i-1},f_{e}^{t},f_{e}^{i+1}), & i=2,3,4 \end{array}\right.$$

where $f_{t}^{i}\left(i\in \left\{1,2,3,4\right\}\right)$ denotes the output features from CIM and represents the fusion of cross-level features from the previous, current, and subsequent feature blocks.

The localization branch incorporates two core components: the Hierarchical Refinement Unit (HRU) and the Adaptive Weighted Adjustment Unit (WAU). Initially, the input feature $f_{e}^{i}$ is processed through the HRU, which utilizes asynchronous convolution techniques to extract features and optimize multi-scale details. To comprehensively capture local features at multiple scales, we construct four parallel branches, each tailored to a specific receptive scale. The first branch employs a single 1×1 convolutional layer with residual connection to preserve critical input information. The subsequent three branches expand the receptive fields to capture broader spatial contexts by employing asymmetric convolutions with kernel sizes of t×1 and 1×t, where t is set to 3, 5, and 7. Specifically, the t×1 convolution enhances sensitivity to vertically oriented structures by extending the kernel height, while the 1 × t convolution captures horizontally oriented details by increasing kernel width. This dual-directional receptive field expansion enables each branch to comprehensively encode contextual information at its respective scale. Following these processing steps, the four branches produce output feature maps $h_{1}$, $h_{2}$, $h_{3}$, and $h_{4}$. The specific computational procedure is shown below:

(7) $$h_{r}=Conv_{1\times t}\left(Conv_{t\times 1}\left(Conv_{1\times 1}\left(f_{e}^{i}\right)\right)\right),\;t=3,5,7$$

where $Conv_{1\times 1}\left(*\right)$ denotes the convolution operation using a 1×1 kernel, while $Conv_{t\times 1}\left(*\right)$ and $Conv_{1\times t}\left(*\right)$ indicate convolutions performed separately with kernels of dimensions t×1 and 1×t, respectively. Subsequently, the outputs $h_{1}$, $h_{2}$, $h_{3}$, and $h_{4}$ generated by the HRU are fed into WAU. For each feature $h_{r}$, a channel attention mechanism independently calculates the channel attention $c_{r}$, enhancing the expressiveness along the channel dimension. The attention maps $c_{r},r\in \left\{1,2,3,4\right\}$ are then used to derive corresponding weights $w_{i}$, which are subsequently applied through element-wise multiplication to their respective feature maps, enabling adaptive weighting and refinement. All weighted feature maps are aggregated through summation to produce the integrated output feature $f_{w}^{i}$. The computational procedure is summarized as follows:

(8) $$f_{w}^{i}=\sum_{r=1}^{4}\frac{\exp(\mathrm{Linear}\left(\mathrm{CA}\left(h_{p}\right)\right))}{\sum_{p=1}^{4}\exp\left(\mathrm{Linear}\left(\mathrm{CA}\left(h_{p}\right)\right)\right)}⊗\mathrm{CA}\left(h_{r}\right)⊗h_{r}$$

where Linear(∗) is a linear layer and CA(∗) denotes channel attention operation. Thereafter, spatial information from $f_{w}^{i}$ is extracted via the spatial attention mechanism, yielding the spatially enhanced feature $f_{m}^{i}$.

The two adjacent branches effectively capture features at different levels. Specifically, the lower-level feature $f_{e}^{i-1}$ is first rescaled to match the spatial dimensions of the current feature $f_{e}^{i}$. In order to enrich the local representations, we obtain the refined feature through the depthwise separable convolution and spatial attention mechanism to realize the accurate segmentation. The process can be formulated as:

(9) $$f_{m}^{i-1}=f_{w}^{i}⊗\mathrm{SA}(DSConv\left(Down_{\times 2}\left(f_{e}^{i-1}\right)\right)\;i\in \left\{2,3,4\right\}$$

where $f_{m}^{i-1}$ is the output of the adjacent lower-level branch and $Down_{\times 2}\left(*\right)$ represents the max-pooling layer with a kernel size of 3×3 and stride of 2. Similarly, the higher-level feature $f_{e}^{i+1}$ employs the same operation to refine the global information, which is formulated as follows:

(10) $$f_{m}^{i+1}=f_{w}^{i}⊗\mathrm{SA}(DSConv\left(Up_{\times 2}\left(f_{e}^{i+1}\right)\right)\;i\in \left\{1,2,3,4\right\}$$

where $f_{m}^{i-1}$ is the output of the adjacent higher-level branch and $Up_{\times 2}\left(*\right)$ is the upsampling operation. Following these operations, we merge the output features from each branch with the current feature. The adjacent lower-level branch provides detailed texture information, whereas higher-level branches introduce global semantic context. The fusion process is described as follows:

(11) $$f_{t}^{i}=\left\{\begin{array}{cc} f_{m}^{i+1}⊕\left(f_{m}^{i}⊗f_{m}^{i+1}\right), & i=1\\ f_{m}^{i-1}⊕\left(f_{m}^{i-1}⊗f_{m}^{i}⊗f_{m}^{i+1}\right)⊕f_{m}^{i+1}, & i=2,3,4 \end{array}\right.$$

In summary, CIM effectively connects and integrates contextual semantic information to facilitate accurate salient object segmentation.

### 3.4. Decoder and Loss Function

To obtain accurate salient objects, we employ the widely used deep supervision strategy during the decoding phase, enabling effective supervision [29] of intermediate salient maps. As shown in Figure 1, the decoder comprises five modules, each consisting of convolution layers followed by a deconvolution layer. Through this design, spatial resolution is progressively recovered, and feature representations are iteratively refined. Ultimately, this decoding process generates a salient projection map $S_{i}$ with dimensions 1×256×256, where pixel values range between 0 and 1.

During training, pixel-level supervision is applied to each decoder stage. We employed a hybrid loss ($L_{total}$) function combining pixel-level weighted binary cross-entropy (BCE) loss with map-level weighted intersection-over-union (IoU) loss. Specifically, the pixel-level weighted BCE loss captures the discrepancy between the predicted and actual masks at each pixel, while the map-level weighted IoU loss evaluates the spatial agreement by calculating the intersection-over-union between the predicted and actual masks. The combined hybrid loss can be formally expressed as:

(12) $$L_{total}=\sum_{i=1}^{5}\left(L_{bce}\left(S_{i},G\right)+L_{iou}\left(S_{i},G\right)\right)$$

where *G* denotes the ground truth, and $L_{bce}$ and $L_{iou}$ represent the BCE loss and IoU loss, respectively.

## 4. Experiments and Results

### 4.1. Experiment Protocol

**(1) Datasets.** To assess the effectiveness of our MCFNet, extensive experiments were carried out on three benchmark ORSI-SOD datasets, i.e., ORSSD [20], EORSSD [37], and ORSI4199 [34]. The ORSSD dataset includes 800 images with pixel-level annotations, split into 600 for training and 200 for testing. The EORSSD dataset, an extension of ORSSD, contains 2000 high-resolution images with corresponding ground truths, typically divided into 1400 training and 600 testing samples. The ORSI4199 dataset is the latest and most challenging dataset in the ORSI-SOD field, comprising 2000 training and 2199 testing images. Its rich diversity and complex scenes pose significant challenges for detection models. For each dataset, training and evaluation are conducted using their designated splits.

**(2) Implementation Details.** Our MCFNet was trained and evaluated on a workstation equipped with an NVIDIA RTX 3090 GPU with 24 GB of memory. All input ORSIs were uniformly resized to 256×256 for both the training and testing phases. To improve model robustness and dataset variability, standard augmentation techniques such as horizontal flipping and rotation were employed. The network was optimized using the Adam optimizer [40], with an initial learning rate of $1\times 10^{-4}$, which was decayed by a factor of 0.1 after 50 epochs. Training was conducted for 100 epochs with a batch size of 16.

**(3) Evaluation Metrics.** We thoroughly evaluated the proposed MCFNet against leading state-of-the-art approaches on three benchmark ORSI-SOD datasets, utilizing six standard evaluation metrics: S-measure ($S_{\alpha }$) [41], Mean Absolute Error (MAE, M), mean E-measures ($E_{\xi }^{mean}$) [42], maximum E-measure ($E_{\xi }^{max}$), mean F-measure ($F_{\beta }^{mean}$) [43], and maximum F-measure ($F_{\beta }^{max}$). Specifically, the S-measure captures the structural similarity between region-aware and object-aware predictions, which can be expressed as:

(13) $$M=\frac{1}{H\times W}\sum_{i=1}^{H}\sum_{j=1}^{W}∥S\left(i,j\right)-G\left(i,j\right)∥,$$

where *S* denotes the predicted salient map, *G* denotes the ground truth, and *H* and *W* denote the height and width, respectively. The MAE quantifies the average pixel-wise discrepancy between predicted saliency maps and ground truth. The mathematical expression is formulated as follows:

(14) $$S_{\alpha }=\alpha S_{o}+\left(1-\alpha \right)S_{r},\;\alpha =0.5$$

where $S_{o}$ and $S_{r}$ denote the object-aware and region-aware structural similarity measures, respectively. Both E-measure and F-measure are evaluated in terms of their mean and maximum values, offering a more complete view of performance. The E-measure combines pixel-level precision and object-level consistency to assess structural coherence and is defined as follows:

(15) $$E_{\xi }=\frac{1}{H\times W}\sum_{i=1}^{H}\sum_{j=1}^{W}\phi FM\left(i,j\right)$$

where ϕFM(i,j) represents the enhanced-alignment matrix. The F-measure offers a harmonic trade-off between precision and recall to reflect the overall segmentation accuracy and is defined as follows:

(16) $$F_{\beta }=\frac{(1+\beta^{2})\;\mathrm{precision}\;\times \;\mathrm{recall}\;}{\beta^{2}\;\mathrm{precision}\;+\mathrm{recall}},\;\beta^{2}=0.3$$

Additionally, we analyzed model performance using precision–recall (PR) curves, where curves closer to the top-right corner (1,1) indicate better performance in terms of both precision and recall.

### 4.2. Comparison with State-of-the-Art Methods

**(1) Competing Methods.** In this study, we present MCFNet and evaluate its effectiveness by benchmarking it against 20 leading methods on three widely used datasets, i.e., ORSSD, EORSSD, and ORSI4199, to comprehensively assess both accuracy and generalization capability. Our evaluation incorporates both qualitative and quantitative analyses. For comparative purposes, we selected eight prominent methods from the natural image salient object detection, including R3Net [44], PoolNet [30], EGNet [13], SUCA [16], U2Net [45], GateNet [14], MINet [46], and PA-KRN [15]. Meanwhile, we compared our approach with twelve specialized ORSI-SOD methods, including DAFNet [37], EMFINet [47], AGNet [48], MJRBM [34], ACCoNet [21], CorrNet [49], BAFS-Net [35], ERPNet [38], AESINet [22], SFANet [50], ADSTNet [51], and LSHNet [17]. To ensure fairness and consistency, all models were trained and tested under identical experimental conditions and parameter configurations. Furthermore, predictions from competing models were generated using publicly available implementations provided by their respective authors, ensuring the reliability and reproducibility of our results.

**(2) Quantitative Comparison.** Table 1 presents a comprehensive quantitative comparison of our method against 20 state-of-the-art approaches on the EORSSD, ORSSD, and ORSI4199 datasets. On the ORSSD dataset, our proposed method achieved superior overall performance, ranking first in five evaluation metrics and second in the remaining metric. Notably, PA-KRN previously demonstrated the highest performance among NSI-SOD methods. However, our method outperforms PA-KRN, with significant improvements of 2.52%, 1.80%, 1.60%, 3.93%, and 3.51% in $S_{\alpha }$, $E_{\xi }^{mean}$, $E_{\xi }^{max}$, $F_{\beta }^{mean}$, and $F_{\beta }^{max}$, respectively. While our approach shows slightly lower performance in the S-measure metric compared to the leading ORSI-SOD method, LSHNet, it achieves notable enhancements in all other five metrics, particularly improvements of 0.30% and 0.18% in $E_{\xi }^{mean}$ and $F_{\beta }^{mean}$, respectively.

On the larger EORSSD dataset, our method also achieved outstanding performance. Compared with the PA-KRN, our approach demonstrated improvements of 2.45%, 4.24%, 1.59%, 2.10%, and 2.00% in terms of $F_{\beta }^{max}$, $F_{\beta }^{mean}$, $E_{\xi }^{max}$, $E_{\xi }^{mean}$, and $S_{\alpha }$, respectively. When compared to the second-best method, LSHNet, our method demonstrated improvements of 0.42% and 0.80% in terms of $E_{\xi }^{mean}$ and $F_{\beta }^{mean}$, respectively. Moreover, we observed a substantial improvement in MAE, achieving a notable reduction of 9.38% compared to LSHNet, demonstrating the superiority of our proposed method.

**Figure 4:**
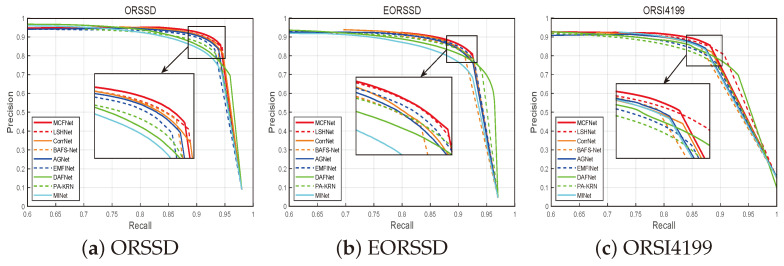
Precision–recall curves of eight advanced models evaluated on three datasets.

On the challenging ORSI4199 dataset, our method exhibited state-of-the-art performance, achieving the highest rank across all six evaluation metrics. Specifically, our model substantially surpassed PA-KRN by 4.03%, 3.04%, 2.45%, 5.30%, and 3.89% in terms of $S_{\alpha }$, $E_{\xi }^{mean}$, $E_{\xi }^{max}$, $F_{\beta }^{mean}$, and $F_{\beta }^{max}$, respectively. Compared to the second-best model, our approach improved critical metrics of M and $S_{\alpha }$ by 3.0% and 0.10%, respectively. Figure 4 illustrates the PR curves on the three benchmark datasets, demonstrating that our MCFNet achieves superior precision and recall. Notably, the PR curves for our method are positioned closest to the upper-right corner, indicating consistently higher performance compared with all of the other evaluated methods.

Overall quantitative analyses confirm that our proposed model achieves new state-of-the-art performance on the ORSI-SOD task, striking a favorable balance between accuracy and computational efficiency. Furthermore, a comparative evaluation between specialized ORSI-SOD and general NSI-SOD methods reveals a significant advantage of ORSI-specific models. These findings underscore the critical importance and urgency of developing dedicated approaches tailored explicitly for remote sensing imagery.

**Figure 5:**
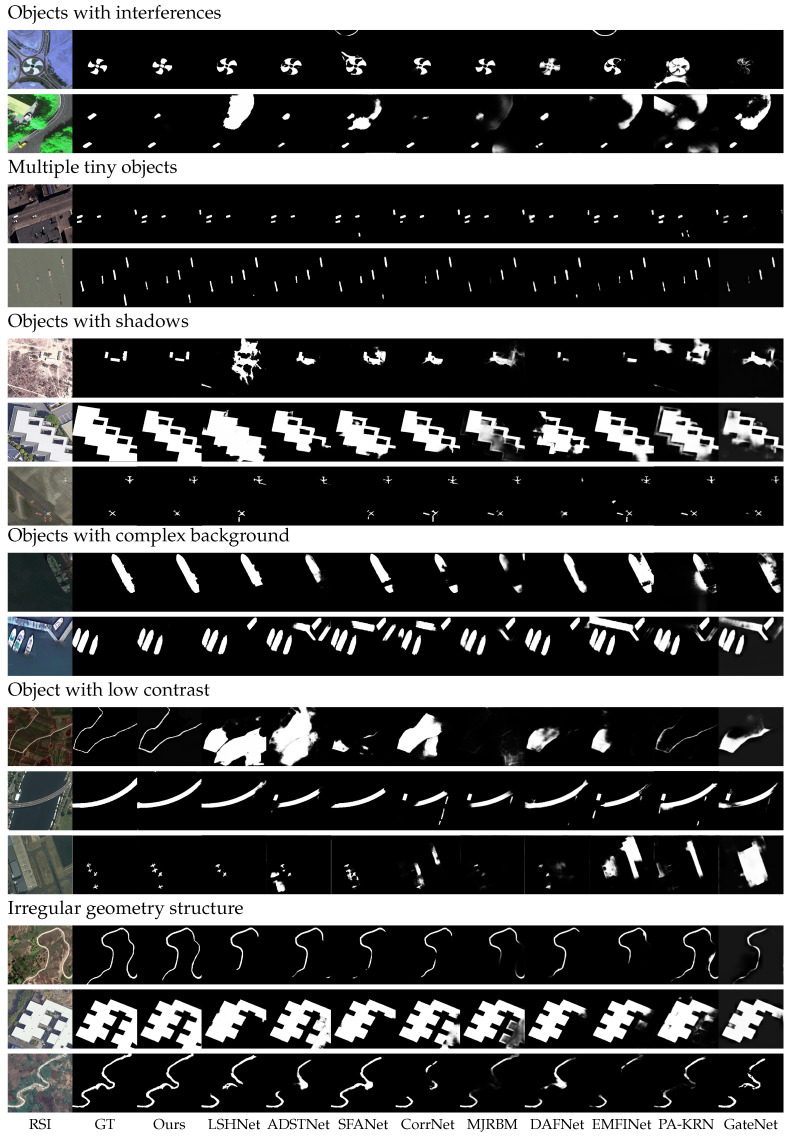
Visual comparisons with nine representative state-of-the-art methods, including two NSI-SOD methods and seven ORSI-SOD methods.

**(3) Qualitative Analysis.** In Figure 5, we present representative and challenging ORSI examples to qualitatively compare MCFNet with nine state-of-the-art approaches. These scenarios encompass cases such as interference from surrounding elements, clusters of small objects, shadow-covered targets, complex background textures, low-contrast regions, and irregularly shaped structures. In remote sensing imagery, non-salient regions frequently cause distractions, which can result in inaccurate detection results. As illustrated in the first and second rows of Figure 5, other methods such as LSHNet, CorrNet, and EMFINet exhibit limitations, either fragmenting target shapes or misidentifying background objects as salient targets. In contrast, our proposed method effectively addresses these challenges by preserving the integrity of salient objects while mitigating interference. MCFNet accurately differentiates salient targets from distractors by leveraging contextual modeling. Furthermore, the integration of SAM guides the model to focus on discriminative features relevant to the salient target while suppressing the influence of non-salient objects. This design enables our model to robustly isolate and highlight salient objects even in complex scenes with potential interferences.

In ORSI, the variability in shooting distances and angles often results in objects exhibiting tiny features, particularly in scenes containing multiple small targets, which presents a significant challenge for SOD. The third and fourth rows of Figure 5 illustrate qualitative results in such complex scenarios. As evident from the visualizations, existing state-of-the-art methods (i.e., LSHNet, ADSTNet, and GateNet) often lead to omissions. Meanwhile, methods such as SFANet and PA-KRN erroneously identify non-target regions as salient objects. In contrast, our method demonstrates superior performance in multiple tiny object scenarios, accurately detecting all targets without false positives or omissions.

Shadows present a significant challenge in SOD, as they are often misclassified as salient regions, leading to inaccurate detection. As illustrated in the fifth and sixth rows of Figure 5, many existing methods erroneously highlight shadowed areas, which affects the completeness of target detection. In contrast, our proposed approach accurately identifies target objects while preserving their structural integrity, effectively mitigating the interference of redundant shadow regions.

Effectively mitigating background interference is a critical challenge in salient object detection, particularly in complex environments. As shown in row 7 of Figure 5, all comparative methods, except for our proposed MCFNet, fail to suppress background interference, resulting in excessive background inclusion and incomplete object detection. In row 8, only our method successfully isolates the salient target from the background, achieving precise and robust detection. These results strongly demonstrate the effectiveness and resilience of our model in handling complex background conditions.

Low-contrast object detection remains a challenging aspect of SOD, as salient targets often exhibit visual similarity to the background, making them difficult to distinguish. As shown in the low-contrast bridge detection example in row 9 of Figure 5, existing methods struggle to accurately delineate the complete structure of the bridge, resulting in incomplete predictions. Similarly, in the scenario presented in row 10, methods such as ADSTNet, EMFINet, and PA-KRN fail to correctly identify the true target object, mistakenly segmenting visually similar background regions instead. In contrast, our method not only accurately detects the target but also precisely delineates its boundaries, demonstrating superior robustness in low-contrast settings.

Salient objects in remote sensing images often exhibit complex and irregular topologies, making accurate edge delineation particularly challenging. In practice, methods such as LSHNet, SFANet, and MJRBM frequently fail to capture the full extent of rivers and buildings, producing fragmented or jagged boundaries that poorly represent the true geometry of the targets. In contrast, our method demonstrates superior performance by accurately detecting rivers with well-defined contours and preserving the structural integrity of buildings. This advantage stems from the integration of a semantic-aware attention mechanism and an adjacent contextual information fusion strategy. The semantic-aware attention mechanism enhances detection completeness by guiding the model to focus more holistically on key target features. Simultaneously, the contextual interaction strategy significantly improves boundary sharpness and enables the model to more effectively handle irregular geometric structures.

### 4.3. Ablation Experiment

In this study, we conducted a series of ablation experiments to systematically evaluate the effectiveness of each component within MCFNet. Specifically, our analysis focused on four key aspects: (1) the individual contribution of each module, (2) the effectiveness of branches in CIM, (3) the effectiveness of semantic-aware attention, and (4) the model’s sensitivity to different backbone architectures. To ensure experimental fairness, each ablation variant was retrained under identical configurations, and their performance was rigorously evaluated and compared.

**Figure 6:**
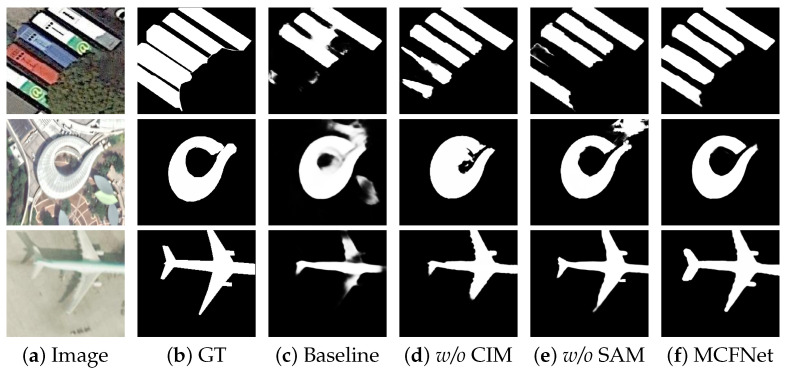
Visual comparisons of the key components. ‘*w*/*o*’ denotes ‘WITHOUT’.

**Effectiveness of the proposed module.** To assess the practical contribution of the proposed modules, we designed four model variants for ablation studies. Variant 1 serves as the baseline model, incorporating the Res2Net backbone and the decoder. Variant 2 integrates SAM into the baseline, while variant 3 includes the CIM. Variant 4 combines both SAM and CIM, representing the full version of our model. Experiments were conducted on the ORSSD and EORSSD datasets, with quantitative results summarized in Table 2. With the integration of SAM, variant 2 achieved notable performance gains on the ORSSD dataset, with improvements of 1.51%, 0.50%, 0.46%, and 0.0019 in terms of $S_{\alpha }$, $E_{\xi }^{max}$, $F_{\beta }^{max}$, and M, respectively. With the addition of SAM, the $F_{\beta }^{max}$ improved by 0.67% and 1.21% on the EORSSD and ORSI4199 datasets, respectively. Similarly, the addition of the CIM results in consistent and significant enhancements across all evaluation metrics. When SAM and CIM are incorporated, the model demonstrates the most substantial performance improvements in $S_{\alpha }$, $E_{\xi }^{max}$, $F_{\beta }^{max}$, and M surpassing the baseline by 1.91%, 0.96%, 1.91%, and 0.0039 on the EORSSD dataset, respectively. These results indicate that each module plays an indispensable role, as removing any one of them results in a noticeable decline in performance. As illustrated in the visual results in Figure 6, the incorporation of SAM can accurately strengthen the localization ability of objects, allowing it to more accurately identify salient regions and suppress background interference. Meanwhile, CIM facilitates effective fusion and interaction between high-level semantic features and low-level texture details, leading to a more coherent and natural delineation of object contours.

**The effectiveness of branches in CIM.** To further assess the contribution of individual branches within the CIM module, we designed two ablated variants: one excluding the adjacent branches (*w*/*o* AB) and the other omitting the current branch (*w*/*o* CB). As shown in Table 3, both variants exhibit noticeable drops in performance relative to the full CIM configuration. These results underscore the importance of both branch types in boosting the overall effectiveness of the model. These findings confirm the effectiveness of the dual-branch design in facilitating cross-scale information fusion and underscore the importance of incorporating both current and neighboring contextual features. Specifically, removal of the current branch leads to a substantial performance drop on the ORSSD dataset, with declines of 1.03%, 0.68%, and 1.05% in $S_{\alpha }$, $E_{\xi }^{mean}$, and $F_{\beta }^{mean}$ on ORSSD, respectively. A similar trend is observed on the EORSSD dataset, where *w*/*o* CB causes substantial drops of 1.40%, 0.89%, and 0.87% in $S_{\alpha }$, $E_{\xi }^{mean}$, and $F_{\beta }^{mean}$, respectively. The performance on the ORSI4199 dataset drops significantly when either the current branch or adjacent branches are removed, with decreases of 1.08% and 0.67% in $S_{\alpha }$, and 1.20% and 1.05% in $F_{\beta }^{\mathrm{mean}}$, respectively. Although the removal of the adjacent branches also leads to a measurable decline, its effect is less pronounced, further highlighting the critical role of the current branch in maintaining performance.

**Figure 7:**
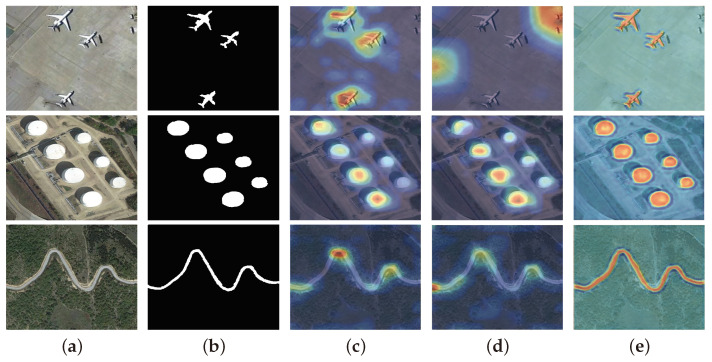
Visual comparisons of semantic-aware attention. (**a**) Optical RSIs. (**b**) Ground truth. (**c**) Self-attention variant. (**d**) Gated attention variant. (**e**) Our MCFNet.

**The effectiveness of semantic-aware attention.** To further assess the effectiveness of semantic-aware attention (SAM), we conducted two variant experiments by replacing semantic-aware attention with either self-attention [52] or gated attention [53]. As shown in Figure 7, the corresponding heat maps offer an intuitive visualization of their respective performances. In the self-attention variant, we observe a more uniform attention distribution across the feature map, reflecting a stronger global capacity for capturing overall contextual information. However, compared with semantic-aware attention, self-attention is less adept at extracting fine-grained features in regions requiring precise semantic cues. This limitation is particularly evident when objects share similar appearances, where self-attention often struggles to differentiate foreground from background, leading to reduced performance. In contrast, the gated attention variant concentrates attention more narrowly through a gating mechanism, thereby highlighting critical features and improving feature selection accuracy. Despite this advantage, gated attention lacks the deeper semantic comprehension inherent to semantic-aware attention. Consequently, when dealing with optical remote sensing images containing complex backgrounds, gated attention underperforms due to its more superficial handling of implicit semantic relationships.

**The sensitivity to various backbones.** In evaluating our module’s sensitivity to different backbone networks, we have chosen three well-known encoders, VGG16, PVTv2, and ResNet-50. For each encoder, we constructed a corresponding variant model (MCFNet-VGG, MCFNet-PVT, and MCFNet-ResNet) that preserves our model’s core structure while substituting the backbone, facilitating a comprehensive assessment of performance. Table 4 provides detailed metrics for each variant. Across all three backbones, our proposed model always has a stable performance, which demonstrates robustness and flexibility. Further analysis revealed that the Res2Net backbone yielded superior results. Consequently, Res2Net was chosen as the default backbone for the final model configuration.

## 5. Conclusions

ORSI-SOD is notably challenging due to the diversity in target number and scale, complex background environments, and irregular topologies. To tackle these issues, we proposed a novel Multi-scale Contextual Fusion Network (MCFNet), comprising a Semantic-Aware Attention Module (SAM) and a Contextual Interconnection Module (CIM). Specifically, SAM introduces preliminary semantic masks to provide accurate semantic guidance during feature extraction, enabling the model to capture long-range dependencies and localize targets robustly. Meanwhile, CIM fuses contextual information across adjacent feature levels through multi-scale refinement, thereby enriching and optimizing texture representations at different scales. This cross-level interaction not only bolsters the model’s ability to discern salient target features but also improves its capacity to suppress background interference, thus increasing robustness. Extensive evaluations and ablation studies on three widely used datasets demonstrate that MCFNet surpasses 20 competing methods by producing more complete and accurate salient regions while maintaining robust detection performance even in complex scenarios.

## Figures and Tables

**Table 1:** Quantitative comparison of our MCFNet with 20 other state-of-the-art methods on EORSSD, ORSSD, and ORSI4199 datasets. The best three results are sequentially marked in red, blue, and green. ‘CN’ is the CNN-based NSI-SOD method, and ‘CR’ is the CNN-based ORSI-SOD method.

Methods	Type	Params	ORSSD						EORSSD						ORSI4199					
		(M)↓	$\;\;S_{\alpha }↑$	M↓	$E_{\xi }^{\mathrm{mean}}↑$	$E_{\xi }^{\max}↑$	$F_{\beta }^{\mathrm{mean}}↑$	$F_{\beta }^{\max}↑$	$\;\;S_{\alpha }↑$	M↓	$E_{\xi }^{\mathrm{mean}}↑$	$E_{\xi }^{\max}↑$	$F_{\beta }^{\mathrm{mean}}↑$	$F_{\beta }^{\max}↑$	$\;\;S_{\alpha }↑$	M↓	$E_{\xi }^{\mathrm{mean}}↑$	$E_{\xi }^{\max}↑$	$F_{\beta }^{\mathrm{mean}}↑$	$F_{\beta }^{\max}↑$
R3Net_18_ [44]	CN	56.2	0.8141	0.0399	0.8681	0.8913	0.7383	0.7456	0.8184	0.0171	0.8294	0.9483	0.6302	0.7498	0.8392	0.0401	0.9021	0.9141	0.8127	0.8250
PoolNet_19_ [30]	CN	53.6	0.8403	0.0358	0.8650	0.9343	0.6999	0.7706	0.8207	0.0210	0.8193	0.9292	0.6406	0.7545	0.8184	0.0573	0.8028	0.8159	0.7332	0.7457
EGNet_19_ [13]	CN	108.1	0.8721	0.0216	0.9013	0.9731	0.7500	0.8332	0.8601	0.0110	0.8775	0.9570	0.6967	0.7880	0.8362	0.0424	0.8916	0.9008	0.8223	0.8368
SUCA_20_ [16]	CN	115.6	0.9285	0.0102	0.9611	0.9698	0.8723	0.8885	0.9126	0.0079	0.9396	0.9644	0.9029	0.8535	0.8294	0.0428	0.8271	0.8340	0.7813	0.7927
U2Net_20_ [45]	CN	44.0	0.9162	0.0166	0.9387	0.9539	0.8492	0.9738	0.9199	0.0076	0.9373	0.9649	0.8329	0.8732	0.8379	0.0391	0.8988	0.9034	0.8201	0.8325
GateNet_20_ [14]	CN	128.6	0.9204	0.0110	0.9560	0.9708	0.8741	0.9035	0.9071	0.0081	0.9364	0.9634	0.8294	0.8646	0.8501	0.0377	0.9155	0.9264	0.8347	0.8489
MINet_20_ [46]	CN	47.6	0.9040	0.0144	0.9454	0.9545	0.8574	0.8761	0.9040	0.0093	0.9346	0.9442	0.8174	0.8344	0.8498	0.0367	0.9098	0.9163	0.8322	0.8473
PA-KRN_21_ [15]	CN	141.1	0.9239	0.0139	0.9620	0.9680	0.8727	0.8890	0.9192	0.0104	0.9536	0.9616	0.8358	0.8639	0.8428	0.0385	0.9121	0.9241	0.8257	0.8432
DAFNet_21_ [37]	CR	29.4	0.9191	0.0113	0.9539	0.9771	0.8511	0.8928	0.9166	0.0060	0.9290	0.9659	0.7842	0.8612	0.8653	0.0344	0.9167	0.9365	0.8244	0.8470
EMFINet_22_ [47]	CR	95.1	0.9432	0.0095	0.9726	0.9813	0.9000	0.9155	0.9319	0.0075	0.9598	0.9712	0.8505	0.8742	0.8591	0.0452	0.9022	0.9116	0.8100	0.8169
AGNet_22_ [48]	CR	24.6	0.9389	0.0091	0.9728	0.9811	0.8956	0.9098	0.9287	0.0067	0.9656	0.9752	0.8516	0.8758	0.8627	0.0337	0.9276	0.9386	0.8536	0.8614
MJRBM_22_ [34]	CR	43.5	0.9193	0.0146	0.9472	0.9631	0.8544	0.8850	0.9180	0.0107	0.9339	0.9631	0.8274	0.8638	0.8582	0.0372	0.9071	0.9343	0.8305	0.8511
ACCoNet_22_ [21]	CR	127.0	0.9437	0.0088	0.9754	0.9796	0.8971	0.9149	0.9290	0.0074	0.9653	0.9727	0.8552	0.8837	0.8675	0.0314	0.9342	0.9412	0.8610	0.8646
CorrNet_22_ [49]	CR	4.1	0.9380	0.0098	0.9764	0.9790	0.9002	0.9129	0.9289	0.0083	0.9646	0.9696	0.8620	0.8778	0.8623	0.0366	0.9206	0.9330	0.8513	0.8560
BAFS-Net_23_ [35]	CR	31.0	0.9378	0.0083	0.9773	0.9820	0.9016	0.9106	0.9250	0.0061	0.9697	0.9729	0.8564	0.8653	0.8661	0.0314	0.9339	0.9399	0.8588	0.8633
ERPNet_23_ [38]	CR	77.2	0.9352	0.0114	0.9604	0.9738	0.8798	0.9036	0.9252	0.0082	0.9366	0.9665	0.8269	0.8743	0.8652	0.0367	0.9167	0.9284	0.8387	0.8538
AESINet_23_ [22]	CR	51.6	0.9455	0.0085	0.9741	0.9814	0.8962	0.9160	0.9347	0.0064	0.9647	0.9757	0.8496	0.8792	0.8702	0.0309	0.9357	0.9423	0.8626	0.8676
SFANet_24_ [50]	CR	25.1	0.9453	0.0077	0.9789	0.9830	0.9063	0.9192	0.9349	0.0058	0.9726	0.9769	0.8680	0.8833	0.8761	0.0292	0.9385	0.9447	0.8659	0.8710
ADSTNet_24_ [51]	CR	62.1	0.9379	0.0086	0.9740	0.9807	0.9042	0.9124	0.9311	0.0065	0.9709	0.9769	0.8716	0.8804	0.8710	0.0318	0.9356	0.9433	0.8653	0.8698
LSHNet_24_ [17]	CR	-	0.9491	0.0075	0.9764	0.9824	0.9054	0.9200	0.9370	0.0064	0.9692	0.9761	0.8643	0.8844	0.8759	0.0299	0.9392	0.9462	0.8690	0.8758
MCFNet (Ours)	CR	29.6	0.9472	0.0075	0.9793	0.9835	0.9070	0.9202	0.9374	0.0058	0.9733	0.9769	0.8712	0.8851	0.8768	0.0290	0.9398	0.9467	0.8695	0.8760

**Table 2:** Ablation analysis measuring the overall contribution of SAM and CIM in MCFNet on the ORSSD, EORSSD, and ORSI4199 datasets. The best results are marked in **bold**.

Method	ORSSD				EORSSD				ORSI4199			
	$\;\;S_{\alpha }↑$	M↓	$E_{\xi }^{\max}↑$	$F_{\beta }^{\max}↑$	$\;\;S_{\alpha }↑$	M↓	$E_{\xi }^{\max}↑$	$F_{\beta }^{\max}↑$	$\;\;S_{\alpha }↑$	M↓	$E_{\xi }^{\max}↑$	$F_{\beta }^{\max}↑$
Baseline	0.9203	0.0112	0.9659	0.8964	0.9198	0.0097	0.9676	0.8685	0.8498	0.0352	0.9267	0.8485
*w*/*o* CIM	0.9342	0.0093	0.9707	0.9105	0.9213	0.0075	0.9701	0.8742	0.8661	0.0336	0.9390	0.8588
*w*/*o* SAM	0.9401	0.0081	0.9739	0.9146	0.9296	0.0069	0.9722	0.8783	0.8683	0.0314	0.9402	0.8655
MCFNet	**0.9472**	**0.0075**	**0.9835**	**0.9202**	**0.9374**	**0.0058**	**0.9769**	**0.8851**	**0.8768**	**0.0290**	**0.9467**	**0.8760**

**Table 3:** Ablation analysis of branch effectiveness in the CIM. THE *w*/*o* denotes ‘WITHOUT’, and the best results are highlighted in **bold**.

Method	ORSSD				EORSSD				ORSI4199			
	$\;\;S_{\alpha }↑$	M↓	$E_{\xi }^{\max}↑$	$F_{\beta }^{\max}↑$	$\;\;S_{\alpha }↑$	M↓	$E_{\xi }^{\max}↑$	$F_{\beta }^{\max}↑$	$\;\;S_{\alpha }↑$	M↓	$E_{\xi }^{\max}↑$	$F_{\beta }^{\max}↑$
*w*/*o* CB	0.9375	0.0088	0.9726	0.8976	0.9245	0.0070	0.9647	0.8637	0.8673	0.0322	0.9316	0.8591
*w*/*o* AB	0.9402	0.0082	0.9741	0.9009	0.9309	0.0064	0.9692	0.8664	0.8709	0.0316	0.9338	0.8604
MCFNet	**0.9472**	**0.0075**	**0.9793**	**0.9070**	**0.9374**	**0.0058**	**0.9733**	**0.8712**	**0.8768**	**0.0290**	**0.9398**	**0.8695**

**Table 4:** Performance evaluation of our MCFNet on various encoder backbones. The best results are marked in **bold**.

Method	ORSSD				EORSSD				ORSI4199			
	$\;\;S_{\alpha }↑$	M↓	$E_{\xi }^{\max}↑$	$F_{\beta }^{\max}↑$	$\;\;S_{\alpha }↑$	M↓	$E_{\xi }^{\max}↑$	$F_{\beta }^{\max}↑$	$\;\;S_{\alpha }↑$	M↓	$E_{\xi }^{\max}↑$	$F_{\beta }^{\max}↑$
MCFNet-VGG	0.9434	0.0080	0.9813	0.9142	0.9328	0.0065	0.9724	0.8819	0.8705	0.0299	0.9411	0.8711
MCFNet-PVT	0.9408	0.0083	0.9802	0.9108	0.9299	0.0066	0.9700	0.8793	0.8683	0.0307	0.9387	0.8684
MCFNet-ResNet	0.9463	0.0078	0.9820	0.9193	0.9351	0.0062	0.9753	0.8842	0.8735	0.0296	0.9436	0.8734
MCFNet(Ours)	**0.9472**	**0.0075**	**0.9835**	**0.9202**	**0.9374**	**0.0058**	**0.9769**	**0.8851**	**0.8768**	**0.0290**	**0.9467**	**0.8760**

## Data Availability

The original contributions presented in the study are included in the article, further inquiries can be directed to the corresponding author.

## References

[1] Deng B., Liu D., Cao Y., Liu H., Yan Z., Chen H. (2024). CFRNet: Cross-Attention-Based Fusion and Refinement Network for Enhanced RGB-T Salient Object Detection. Sensors.

[2] Peng Y., Zhai Z., Feng M. (2024). SLMSF-Net: A Semantic Localization and Multi-Scale Fusion Network for RGB-D Salient Object Detection. Sensors.

[3] Zhao J., Wen X., He Y., Yang X., Song K. (2024). Wavelet-Driven Multi-Band Feature Fusion for RGB-T Salient Object Detection. Sensors.

[4] Chen C., Mo J., Hou J., Wu H., Liao L., Sun W., Yan Q., Lin W. (2024). TOPIQ: A Top-Down Approach From Semantics to Distortions for Image Quality Assessment. IEEE Trans. Image Process..

[5] Huang W., Feng H., Xu H., Liu X., He J., Gan L., Wang X., Wang S. (2025). Surface Vessels Detection and Tracking Method and Datasets with Multi-Source Data Fusion in Real-World Complex Scenarios. Sensors.

[6] Liu S., Shen X., Xiao S., Li H., Tao H. (2025). A Multi-Scale Feature-Fusion Multi-Object Tracking Algorithm for Scale-Variant Vehicle Tracking in UAV Videos. Remote Sens..

[7] Cho D., Park J., Oh T.H., Tai Y.W., So Kweon I. Weakly-and Self-Supervised Learning for Content-Aware Deep Image Retargeting. Proceedings of the IEEE International Conference on Computer Vision.

[8] Zheng J., Quan Y., Zheng H., Wang Y., Pan X. (2023). ORSI Salient Object Detection via Cross-Scale Interaction and Enlarged Receptive Field. IEEE Geosci. Remote Sens. Lett..

[9] Tong N., Lu H., Ruan X., Yang M.H. Salient Object Detection via Bootstrap Learning. Proceedings of the IEEE Conference on Computer Vision and Pattern Recognition.

[10] Song H., Liu Z., Du H., Sun G., Le Meur O., Ren T. (2017). Depth-Aware Salient Object Detection and Segmentation via Multiscale Discriminative Saliency Fusion and Bootstrap Learning. IEEE Trans. Image Process..

[11] Zhou L., Yang Z., Zhou Z., Hu D. (2017). Salient Region Detection Using Diffusion Process on a Two-Layer Sparse Graph. IEEE Trans. Image Process..

[12] Ding L., Wang X., Li D. (2022). Visual Saliency Detection in High-Resolution Remote Sensing Images Using Object-Oriented Random Walk Model. IEEE J. Sel. Top. Appl. Earth Obs. Remote Sens..

[13] Zhao J.X., Liu J.J., Fan D.P., Cao Y., Yang J., Cheng M.M. EGNet: Edge Guidance Network for Salient Object Detection. Proceedings of the IEEE International Conference on Computer Vision.

[14] Zhao X., Pang Y., Zhang L., Lu H., Zhang L. Suppress and Balance: A Simple Gated Network for Salient Object Detection. Proceedings of the European Conference on Computer Vision.

[15] Xu B., Liang H., Liang R., Chen P. Locate Globally, Segment Locally: A Progressive Architecture With Knowledge Review Network for Salient Object Detection. Proceedings of the 25th AAAI Conference on Artificial Intelligence (AAAI 21).

[16] Li J., Pan Z., Liu Q., Wang Z. (2020). Stacked U-Shape Network With Channel-Wise Attention for Salient Object Detection. IEEE Trans. Multimed..

[17] Lee S., Cho S., Park C., Park S., Kim J., Lee S. (2024). LSHNet: Leveraging Structure-Prior With Hierarchical Features Updates for Salient Object Detection in Optical Remote Sensing Images. IEEE Trans. Geosci. Remote Sens..

[18] He J., Zhao L., Hu W., Zhang G., Wu J., Li X. (2023). TCM-Net: Mixed Global–Local Learning for Salient Object Detection in Optical Remote Sensing Images. Remote Sens..

[19] Huo L., Hou J., Feng J., Wang W., Liu J. (2024). Global and multiscale aggregate network for saliency object detection in optical remote sensing images. Remote Sens..

[20] Li C., Cong R., Hou J., Zhang S., Qian Y., Kwong S. (2019). Nested Network with Two-Stream Pyramid for Salient Object Detection in Optical Remote Sensing Images. IEEE Trans. Geosci. Remote Sens..

[21] Li G., Liu Z., Zeng D., Lin W., Ling H. (2022). Adjacent Context Coordination Network for Salient Object Detection in Optical Remote Sensing Images. IEEE Trans. Cybern..

[22] Zeng X., Xu M., Hu Y., Tang H., Hu Y., Nie L. (2023). Adaptive Edge-Aware Semantic Interaction Network for Salient Object Detection in Optical Remote Sensing Images. IEEE Trans. Geosci. Remote Sens..

[23] Liu Z., Zou W., Le Meur O. (2014). Saliency tree: A novel saliency detection framework. IEEE Trans. Image Process..

[24] Yu J.G., Zhao J., Tian J., Tan Y. (2013). Maximal entropy random walk for region-based visual saliency. IEEE Trans. Cybern..

[25] Yuan Y., Li C., Kim J., Cai W., Feng D.D. (2017). Reversion correction and regularized random walk ranking for saliency detection. IEEE Trans. Image Process..

[26] Zhou Y., Huo S., Xiang W., Hou C., Kung S.Y. (2019). Semi-Supervised Salient Object Detection Using a Linear Feedback Control System Model. IEEE Trans. Cybern..

[27] Pan C., Liu J., Yan W.Q., Cao F., He W., Zhou Y. (2021). Salient Object Detection Based on Visual Perceptual Saturation and Two-Stream Hybrid Networks. IEEE Trans. Image Process..

[28] Liang M., Hu X. (2014). Feature selection in supervised saliency prediction. IEEE Trans. Cybern..

[29] Jang S.W., Yan L., Kim G.Y. (2025). Deep Supervised Attention Network for Dynamic Scene Deblurring. Sensors.

[30] Liu J.J., Hou Q., Cheng M.M., Feng J., Jiang J. A Simple Pooling-Based Design for Real-Time Salient Object Detection. Proceedings of the IEEE Conference on Computer Vision and Pattern Recognition.

[31] Ma F., Zhang F., Yin Q., Xiang D., Zhou Y. (2022). Fast SAR Image Segmentation With Deep Task-Specific Superpixel Sampling and Soft Graph Convolution. IEEE Trans. Geosci. Remote Sens..

[32] Ma F., Zhang F., Xiang D., Yin Q., Zhou Y. (2022). Fast Task-Specific Region Merging for SAR Image Segmentation. IEEE Trans. Geosci. Remote Sens..

[33] Luo H., Liang B. (2023). Semantic-Edge Interactive Network for Salient Object Detection in Optical Remote Sensing Images. IEEE J. Sel. Top. Appl. Earth Obs. Remote Sens..

[34] Tu Z., Wang C., Li C., Fan M., Zhao H., Luo B. (2022). ORSI Salient Object Detection via Multiscale Joint Region and Boundary Model. IEEE Trans. Geosci. Remote Sens..

[35] Gu Y., Xu H., Quan Y., Chen W., Zheng J. (2023). ORSI Salient Object Detection via Bidimensional Attention and Full-stage Semantic Guidance. IEEE Trans. Geosci. Remote Sens..

[36] Li G., Bai Z., Liu Z., Zhang X., Ling H. (2023). Salient object detection in optical remote sensing images driven by transformer. IEEE Trans. Image Process..

[37] Zhang Q., Cong R., Li C., Cheng M.M., Fang Y., Cao X., Zhao Y., Kwong S. (2021). Dense Attention Fluid Network for Salient Object Detection in Optical Remote Sensing Images. IEEE Trans. Image Process..

[38] Zhou X., Shen K., Weng L., Cong R., Zheng B., Zhang J., Yan C. (2023). Edge-Guided Recurrent Positioning Network for Salient Object Detection in Optical Remote Sensing Images. IEEE Trans. Cybern..

[39] Vaswani A., Shazeer N., Parmar N., Uszkoreit J., Jones L., Gomez A.N., Kaiser L., Polosukhin I. Attention is All You Need. Proceedings of the International Conference on Neural Information Processing Systems.

[40] Kingma D.P., Ba J. Adam: A Method for Stochastic Optimization. Proceedings of the International Conference on Learning Representations.

[41] Fan D.P., Cheng M.M., Liu Y., Li T., Borji A. Structure-Measure: A New Way to Evaluate Foreground Maps. Proceedings of the IEEE International Conference on Computer Vision.

[42] Fan D.P., Gong C., Cao Y., Ren B., Cheng M.M., Borji A. Enhanced-Alignment Measure for Binary Foreground Map Evaluation. Proceedings of the International Joint Conference on Artificial Intelligence.

[43] Achanta R., Hemami S., Estrada F., Susstrunk S. Frequency-Tuned Salient Region Detection. Proceedings of the IEEE Conference on Computer Vision and Pattern Recognition.

[44] Deng Z., Hu X., Zhu L., Xu X., Qin J., Han G., Heng P.A. R3Net: Recurrent Residual Refinement Network for Saliency Detection. Proceedings of the 27th International Joint Conference on Artificial Intelligence.

[45] Qin X., Zhang Z., Huang C., Dehghan M., Zaiane O.R., Jagersand M. (2020). U2-Net: Going deeper with nested U-structure for salient object detection. Pattern Recognit..

[46] Pang Y., Zhao X., Lu H. Multi-Scale Interactive Network for Salient Object Detection. Proceedings of the IEEE Conference on Computer Vision and Pattern Recognition.

[47] Zhou X., Shen K., Liu Z., Gong C., Zhang J., Yan C. (2022). Edge-Aware Multiscale Feature Integration Network for Salient Object Detection in Optical Remote Sensing Images. IEEE Trans. Geosci. Remote Sens..

[48] Lin Y., Sun H., Liu N., Bian Y., Cen J., Zhou H. Attention Guided Network for Salient Object Detection in Optical Remote Sensing Images. Proceedings of the International Conference on Artificial Neural Networks.

[49] Li G., Liu Z., Bai Z., Lin W., Ling H. (2022). Lightweight Salient Object Detection in Optical Remote Sensing Images via Feature Correlation. IEEE Trans. Geosci. Remote Sens..

[50] Quan Y., Xu H., Wang R., Guan Q., Zheng J. (2024). ORSI Salient Object Detection via Progressive Semantic Flow and Uncertainty-Aware Refinement. IEEE Trans. Geosci. Remote Sens..

[51] Zhao J., Jia Y., Ma L., Yu L. (2024). Adaptive Dual-Stream Sparse Transformer Network for Salient Object Detection in Optical Remote Sensing Images. IEEE J. Sel. Top. Appl. Earth Obs. Remote Sens..

[52] Gu Y., Wang L., Wang Z., Liu Y., Cheng M.M., Lu S.P. Pyramid Constrained Self-Attention Network for Fast Video Salient Object Detection. Proceedings of the AAAI Conference on Artificial Intelligence.

[53] Schlemper J., Oktay O., Schaap M., Heinrich M., Kainz B., Glocker B., Rueckert D. (2019). Attention gated networks: Learning to leverage salient regions in medical images. Med. Image Anal..

